# Association between family history and lung cancer risk among Chinese women in Singapore

**DOI:** 10.1038/s41598-021-00929-9

**Published:** 2021-11-08

**Authors:** Xin Yin, Cheryl Pui Yi Chan, Adeline Seow, Wai-Ping Yau, Wei Jie Seow

**Affiliations:** 1 Saw Swee Hock School of Public Health, National University of Singapore and National University Health System, 12 Science Drive 2, Singapore, 117549 Singapore; 2 Department of Pharmacy, Faculty of Science, National University of Singapore, Singapore, 117559 Singapore; 3 Department of Medicine, Yong Loo Lin School of Medicine, National University of Singapore and National University Health System, Singapore, 117597 Singapore

**Keywords:** Risk factors, Cancer epidemiology, Lung cancer

## Abstract

Risk factors of lung cancer unrelated to smoking are not well-studied, especially among women. Family history has been shown to play a role in predisposing individuals to lung cancer, but this relationship has not been investigated in the Southeast Asian population. A total of 1159 women were recruited in a case–control study conducted in public hospitals in Singapore from 2005 to 2008. After excluding participants with incomplete family history information, 374 cases and 785 controls remained in the final analysis. Adjusted odds ratios (aORs) and 95% confidence intervals (CIs) were calculated using logistic regression, adjusting for potential confounders. Overall, family history of lung cancer was associated with a higher risk for lung cancer (aOR 2.08, 95% CI 1.25–3.47). When stratified by smoking status, a significant association was observed among never-smokers (aOR 2.78, 95% CI 1.57–4.90). Further stratification by fruit consumption identified a significant association between family history of lung cancer and higher risk of lung cancer among never-smokers who had low fruit consumption (aOR 3.09, 95% CI 1.37–7.01). Our findings suggest that family history of lung cancer is a significant risk factor for lung cancer in Singaporean Chinese women, especially among never-smokers.

## Introduction

Lung cancer is the most common cancer in men and the third most common cancer in women^1^, with a high global disease burden^1,2^. The risk factors for lung cancer are numerous, and each subtype of lung cancer (e.g. squamous cell carcinoma, adenocarcinoma) has different risk profiles^3,4^. Indubitably, most lung cancer cases today are attributable to cigarette smoking, which increases lung cancer risk by five to 20-fold compared with never-smokers^5,6^. In Singapore, lung cancer is among the top cancer types by incidence and mortality not only for males, but also for females despite their very low smoking prevalence at around 4%^7,8^. Hence, it is important to identify potential risk factors other than smoking to explain the high lung cancer prevalence among Singaporean women.

Family history has been shown to play a role in predisposing individuals to lung cancer^9,10^. Segregation analyses, linkage analysis and genome-wide association studies provide additional evidence of familial aspects to lung cancer risk^11–13^. Previous meta-analyses and pooled analysis provided strong evidence that family history of lung cancer is associated with an increased risk of the disease, reporting odds ratios (ORs) ranging from 1.51 to 2.79^14,15^. A similar trend was found among women with a family history of any cancers as well^16^. In general, ORs are higher in meta-analyses conducted in China than in meta-analyses that included data from other regions. However, the association between family history and lung cancer has not yet been evaluated in Singapore. This study, therefore, aimed to evaluate the association between family history and lung cancer, including its subtypes, among Singaporean Chinese women. Potential effect modifications by other risk factors such as smoking and fruit consumption for lung cancer were also investigated.

## Materials and methods

### Study population

Data for the present analysis was obtained from the Genes and Environment in Lung Cancer (GEL) study, a case–control study conducted in public hospitals from 2005 to 2008. More details on this study have been described previously^17,18^. Briefly, cases were Chinese females enrolled in the study selected based on a diagnosis of primary lung carcinoma and were interviewed within 3 months of diagnosis. Based on these eligibility criteria, 399 patients were identified. In this study, 17 cases were excluded due to incomplete first-degree family history information, 8 cases were later excluded due to incomplete covariates information, leaving 374 cases included in this analysis (Supplementary Fig. 1). Diagnosis of primary lung cancer was ascertained either through histologic or cytologic reports (358 cases) or through radiologic reports (16 cases) with metastatic cancer to the lung from other sites was ruled unlikely.

Controls were hospital-based, frequency matched to cases based on age, date of admission and site of enrolment. They were chosen from a wide range of diagnoses. Patients with cancer or respiratory disease history were excluded. The participation rate was 85.4% from 962 eligible controls identified. In all, 822 subjects were recruited as controls, but 7 were excluded (one was reclassified as a case, while six were later diagnosed with other malignancies). In this study, 21 controls were excluded due to incomplete first-degree family history information, a further 9 controls were excluded due to missing data in other variables, leaving a total of 785 controls included in this analysis (Supplementary Fig. 1).

The study was approved by the National University of Singapore Institutional Review Board, and the Ethics Committees of institutions where the interviews were conducted. Written informed consent was sought from all participants prior to their participation in the study.

### Demographics and family history information

Information was obtained from cases and controls via in-person interviews conducted by trained interviewers. Although interviewers were not blinded to case or control status, interviews were recorded and randomly selected for review to monitor possible interviewer bias. A structured questionnaire was used to collect demographic information including, but not limited to, age, smoking history, environmental tobacco smoke (ETS) exposure, dietary habits and family history of cancer (lung cancer, overall cancer). Family history information was derived from three questions in the questionnaire: (1) Has anyone in your family ever had cancer? (A. Yes. B. No); (2) If yes, was it your…? (A. Parent, sibling or child. B. Husband, or husband’s family. C. Other relative and specify); (3) What type of cancer was it? (A. Lung. B. Other and specify). A positive family history of lung cancer was defined as having a first-degree relative (parent, sibling or child) with lung cancer, and the family history of all cancer was defined as having a first-degree relative (parent, sibling or child) with any kind of cancer, including lung cancer and the other cancers (leukemia and myeloma were also included). ETS exposure was defined as environmental tobacco smoke exposure at home more than once per week and/or environmental tobacco smoke exposure at work. History of respiratory disease was defined as having a medical history of tuberculosis, childhood pneumonia, asthma, chronic bronchitis or emphysema. Food consumption was calculated as the product of average frequency per week and number of standard servings. The cut-off value was determined by the median weekly consumption among controls.

### Statistical analysis

The normality of continuous variables was tested using Shapiro–Wilk test, and all continuous variables are non-normally distributed. To test if baseline characteristics of cases and controls differed, Fisher’s exact test was used for categorical variables and the Mann–Whitney *U* test was used for non-normally distributed continuous variables.

Using logistic regression, we estimated ORs and 95% confidence intervals (CIs) for each association examined. ORs were adjusted (aOR) for potential confounders, which include age, type of dwelling (categorical variable, including 1–3 rooms flat, 4 rooms or more rooms flat, private property and others), years of education (categorical variable, including no formal education, ≤ 6 years, and ≥ 7 years), ETS (yes or no), body mass index (BMI, continuous), history of respiratory disease (yes or no), meat consumption (continuous), fruit consumption (continuous), vegetable consumption (continuous), as well as smoking status (never-smoker or ever-smoker), years of smoking (continuous) and the number of cigarettes per day (continuous). We compared the family history of first-degree relatives of lung cancer cases with that of controls. Subjects were also stratified based on smoking status (never-smoker or ever-smoker) and histologic subtypes (non-small cell lung cancer (NSCLC), which was further stratified as adenocarcinoma and non-adenocarcinoma NSCLC, small cell lung cancer (SCLC), neuroendocrine carcinoma), according to the International Classification of Diseases for Oncology (ICD-O) (Supplementary Table 1). All potential interaction effects with family history of lung/any cancer on the risk of lung cancer were tested using a product term. The analysis was stratified if the interaction p-value was statistically significant.

**Table 1:** Baseline characteristics of the study population (Chinese females).

	Cases (n = 374)	Controls (n = 785)	*P*-value^a^
**Age, years [median (Q1–Q3)]**	68.0 (58.8–75.5)	67.0 (57.3–74.8)	0.16
**Smoking status (n (%))**			< 0.001
Never-smoker	251 (67.1%)	693 (88.3%)	
Ever-smoker	123 (32.9%)	92 (11.7%)	
Average cigarettes per day [median (Q1–Q3)]	7.0 (3.0–12.0)	5.0 (2.0–10.5)	0.028
Average smoking duration, years [median (Q1–Q3)]	44.0 (29.0–57.0)	29.0 (6.0–52.0)	0.0018
Body mass index (BMI), kg/m^2^ [median (Q1–Q3)]	21.6 (19.4–24.8)	23.3 (20.8–26.7)	< 0.001
**Food intake, servings per week [median (Q1–Q3)]**			
Meat consumption^b^	11.2 (6.1–18.6)	13.8 (7.6–23.0)	< 0.001
Fruit consumption^c^	4.9 (1.6–9.3)	7.4 (2.8–14.0)	< 0.001
Vegetable consumption^d^	14.4 (9.0–24.3)	17.3 (9.4–29.7)	0.0047
**Type of dwelling [n (%)]**			0.11
Flat, 1 room–3 rooms	120 (32.1%)	270 (34.4%)	
Flat, 4 rooms or more	185 (49.5%)	412 (52.5%)	
Private property	66 (17.6%)	99 (12.6%)	
Others	3 (0.8%)	4 (0.5%)	
Education [n (%)]			0.67
No formal education	149 (39.6%)	311 (39.8%)	
≤ 6 years	112 (32.2%)	253 (30.0%)	
> 6 years	113 (28.2%)	221 (30.2%)	
**History of respiratory disease**^**e**^** [n (%)]**			0.021
Absent	313 (83.7%)	693 (88.3%)	
Present	61 (16.3%)	92 (11.7%)	
**Family history of cancer [n (%)]**			0.0090
None	258 (69.0%)	608 (77.4%)	
Present-lung cancer	37 (9.9%)	42 (5.4%)	
Present-other cancers	79 (21.1%)	135 (17.2%)	
**Environmental tobacco smoke (ETS) exposure**^**f**^** [n (%)]**			0.20
Absent	116 (31.0%)	264 (33.6%)	
Present	258 (69.0%)	521 (66.4%)	
**Histologic type**^**g**^** [n (%)]**			N/A
Non-small cell lung cancer (NSCLC)	309 (82.6%)	–	
Adenocarcinoma	229 (74.1%)	–	
Squamous cell carcinoma	28 (9.1%)	–	
Large cell carcinoma	4 (1.3%)	–	
Unspecified NSCLC	48 (15.5%)	–	
Small cell	18 (4.8%)	–	
Neuroendocrine carcinoma	5 (1.3%)	–	
Other lung cancer	26 (7.0%)	–	
No histological or cytological data^h^	16 (4.3%)	–	

^a^P-values were obtained using Fisher’s exact test for categorical variables and Mann–Whitney U test for continuous variables.

^b^Fish, chicken, pork, duck, prawns, squid and beef.

^c^Bananas, papayas, apples, oranges, pineapples, watermelons, mangoes, starfruit, jackfruit, plum, cantaloupe, dried prunes, fresh fruit juice and canned peaches.

^d^Wong-nga-pak (Chinese napa cabbage), pak choy (Chinese cabbage), kai lan (Chinese kale), head cabbage, cauliflower, kai choy (Chinse mustard), choy sum (Chinese flowering cabbage), kang kong (water convolvulus), sai yong choy (watercress), por choy (spinach), sang choy (Chinese lettuce), tomatoes, broccoli, French beans, string (long) beans, snow peas, ladies’ fingers, carrot (red) and sweet potato.

^e^Tuberculosis, childhood pneumonia, asthma, chronic bronchitis or emphysema.

^f^ETS exposure is defined as ETS exposure at home more than once per week and/or ETS exposure at work.

^g^Classification based on ICD-O-3 codes detailed in Supplementary Table 1, unless otherwise specified.

^h^Diagnosis of primary lung cancer in these subjects were based on radiological examination and after ruling out of the possibility of malignancy from another primary site.

STATA version 16.0 (Stata Corporation, College Station, Texas, USA) was used for the data analyses. All tests were two-sided, and p-values of less than 0.05 were considered to be statistically significant. 

## Results

The study population consists of Chinese females in Singapore. Smoking was more prevalent among lung cancer cases (*P* < 0.001, Table 1). There were more ever-smokers among cases (32.9%) than controls (11.7%). In addition, ever-smoking cases were more likely to have smoked for a longer period of time (median: 44.0 years vs. 29.0 years, *P* = 0.0018), and a higher number of cigarettes per day than controls (7.0 cigarettes/day vs. 5.0 cigarettes/day, *P* = 0.0278). Cases were more likely than controls to have a history of respiratory disease, family history of cancer, lower BMI, and consumed less meat, fruits and vegetables (Table 1).

After adjusting for potential confounders, family history of lung cancer was more strongly associated with a higher lung cancer risk (aOR 2.08, 95% CI 1.25–3.47) than having a family history of any cancer (aOR 1.59, 95% CI 1.17–2.16), as compared to those with no family history (Table 2). When stratified by smoking status, the association between family history of lung cancer and the lung cancer risk was evident among never-smokers (aOR 2.78, 95% CI 1.57–4.90), but not among ever-smokers (aOR 0.67, 95% CI 0.22–2.04). On the other hand, the association between family history of any cancer and lung cancer was comparable for never-smokers (aOR 1.67, 95% CI 1.19–2.35) and ever-smokers (aOR 1.33, 95% CI 0.64–2.78), albeit not statistically significant. The interaction between family history of lung cancer and smoking status (*P* = 0.021) on lung cancer risk was statistically significant after adjusting for the other variables.

**Table 2:** Odds ratio (OR) and 95% confidence interval (CI) of the association between family history and lung cancer, stratified by smoking status of women and type of cancer in first-degree relatives.

Family history (FH) of cancer in first-degree relatives	Cases	Controls	Crude OR	Adjusted OR
	n (%)	n (%)	(95% CI)	(95% CI)
**All women**				
Lung cancer				
	(n = 295)	(n = 650)		
FH absent	254 (87.5%)	608 (93.5%)	1.00 (ref.)	1.00 (ref.)
FH present	35 (12.5%)	42 (6.5%)	**2.08 (1.30–3.30)**	**2.08 (1.25–3.47)**^**a**^
All cancer				
	(n = 374)	(n = 785)		
FH absent	258 (69.0%)	608 (77.5%)	1.00 (ref.)	1.00 (ref.)
FH present	116 (30.0%)	177 (22.5%)	**1.54 (1.17–2.03)**	**1.59 (1.17–2.16)**^**a**^
**Never-smokers only**				
Lung cancer				
	(n = 193)	(n = 569)		
FH absent	164 (85.0%)	535 (94.0%)	1.00 (ref.)	1.00 (ref.)
FH present	29 (15.0%)	34 (6.0%)	**2.78 (1.65–4.71)**	**2.78 (1.57–4.90)**^**b**^
All cancer				
	(n = 251)	(n = 693)		
FH absent	164 (65.3%)	535 (77.2%)	1.00 (ref.)	1.00 (ref.)
FH present	87 (34.7%)	158 (22.8%)	**1.80 (1.31–2.46)**	**1.67 (1.19–2.35)**^**b**^
**Ever-smokers only**				
Lung cancer				
	(n = 102)	(n = 81)		
FH absent	94 (92.2%)	73 (90.1%)	1.00 (ref.)	1.00 (ref.)
FH present	8 (7.8%)	8 (9.9%)	0.78 (0.28–2.17)	0.67 (0.22–2.04)^c^
All cancer				
	(n = 123)	(n = 92)		
FH absent	94 (76.4%)	73 (79.3%)	1.00 (ref.)	1.00 (ref.)
FH present	29 (23.6%)	19 (20.7%)	1.19 (0.62–2.28)	1.33 (0.64–2.78)^c^

^a^Adjusted for age, type of dwelling, years of education, ETS exposure, BMI, history of respiratory disease, meat consumption, fruit consumption, vegetable consumption, and smoking status.

^b^Adjusted for age, type of dwelling, years of education, ETS exposure, BMI, history of respiratory disease, meat consumption, fruit consumption, and vegetable consumption.

^c^Adjusted for age, type of dwelling, years of education, ETS exposure, BMI, history of respiratory disease, meat consumption, fruit consumption, vegetable consumption, as well as years of smoking and number of cigarettes per day. Four subjects with family history of lung cancer and five subjects with family history of all cancer had missing data on years of smoking and number of cigarettes per day. Bold values refer to statistically significant results with*P*<0.05.

When stratified by the histologic type of lung cancer, family history of lung cancer was significantly associated with NSCLC (aOR 2.07, 95% CI 1.21–3.56) (Table 3). This association was stronger among never-smokers (aOR 2.77, 95% CI 1.53–5.00). The adenocarcinoma results were similar to the NSCLC results since adenocarcinoma made up 77.2% of NSCLCs. Among the adenocarcinoma patients, the positive association remained among both the overall population (aOR 1.90, 95% CI 1.06–3.39) and never-smokers (aOR 2.39, 95% CI 1.26–4.51), as compared to those without family history of lung cancer. For non-adenocarcinoma NSCLC, small cell lung cancer and neuroendocrine carcinoma, a larger proportion of cases than controls reported a family history of lung cancer (Supplementary Table 2). Nonetheless, due to the small number of these cases with family history (fewer than 10), ORs were not calculated because of the limited power.

**Table 3:** Odds ratio (OR) and 95% confidence interval (CI) of the association between family history and lung cancer risk, stratified by histologic type of lung cancer and fruit consumption.

Family history (FH) of cancer in first-degree relatives	All women				Never-smokers only			
	Cases	Controls	Crude OR	Adjusted OR (95% CI)^a^	Cases	Controls	Crude OR	Adjusted OR (95% CI)^b^
	n (%)	n (%)	(95% CI)		n (%)	n (%)	(95% CI)	
**Non-small cell lung cancer (NSCLC)**								
Lung cancer								
	(n = 237)	(n = 650)			(n = 161)	(n = 569)		
FH absent	207 (87.3%)	608 (93.5%)	1.00 (ref.)	1.00 (ref.)	136 (84.5%)	535 (94.0%)	1.00 (ref.)	1.00 (ref.)
FH present	30 (12.7%)	42 (6.5%)	**2.10 (1.28–3.44)**	**2.07 (1.21–3.56)**	25 (15.5%)	34 (6.0%)	**2.89 (1.67–5.01)**	**2.77 (1.53–5.00)**
**Adenocarcinoma (77.2% of NSCLCs)**								
Lung cancer								
	(n = 183)	(n = 650)			(n = 139)	(n = 569)		
FH absent	160 (87.4%)	608 (93.5%)	1.00 (ref.)	1.00 (ref.)	120 (86.3%)	535 (94.0%)	1.00 (ref.)	1.00 (ref.)
FH present	23 (12.6%)	42 (6.5%)	**2.08 (1.22–3.56)**	**1.90 (1.06–3.39)**	19 (13.7%)	34 (6.0%)	**2.49 (1.37–4.52)**	**2.39 (1.26–4.51)**
**Low fruit consumption (≤ 7.36 servings per week)**^**c**^								
Lung cancer								
	(n = 194)	(n = 340)			(n = 119)	(n = 290)		
FH absent	172 (88.7%)	322 (94.7%)	1.00 (ref.)	1.00 (ref.)	102 (85.7%)	277 (95.5%)	1.00 (ref.)	1.00 (ref.)
FH present	22 (11.3%)	18 (5.3%)	**2.29 (1.19–4.38)**	1.98 (0.97–4.06)	17 (14.3%)	13 (4.5%)	**3.56 (1.67–7.57)**	**3.09 (1.37–7.01)**
All cancer								
	(n = 243)	(n = 393)			(n = 151)	(n = 338)		
FH absent	172 (70.8%)	322 (81.9%)	1.00 (ref.)	1.00 (ref.)	102 (67.5%)	277 (81.9%)	1.00 (ref.)	1.00 (ref.)
FH present	71 (29.2%)	71 (18.1%)	**1.87 (1.28–2.73)**	**1.88 (1.24–2.86)**	49 (32.5%)	61 (18.1%)	**2.18 (1.41–3.38)**	**1.99 (1.24–3.19)**
**High fruit consumption (> 7.36 servings per week)**^**c**^								
Lung cancer								
	(n = 101)	(n = 310)			(n = 74)	(n = 279)		
FH absent	86 (85.2%)	286 (92.3%)	1.00 (ref.)	1.00 (ref.)	62 (83.8%)	258 (92.5%)	1.00 (ref.)	1.00 (ref.)
FH present	15 (14.8%)	24 (7.7%)	**2.08 (1.04–4.14)**	**2.24 (1.05–4.77)**	12 (16.2%)	21 (7.5%)	**2.38 (1.11–5.09)**	**2.58 (1.14–5.84)**
All cancer								
	(n = 131)	(n = 392)			(n = 100)	(n = 355)		
FH absent	86 (65.7%)	286 (73.0%)	1.00 (ref.)	1.00 (ref.)	62 (62.0%)	258 (72.7%)	1.00 (ref.)	1.00 (ref.)
FH present	45 (34.3%)	106 (27.0%)	1.41 (0.92–2.16)	1.32 (0.82–2.10)	38 (38.0%)	97 (27.3%)	**1.63 (1.02–2.60)**	1.39 (0.84–2.30)

^a^Adjusted for age, type of dwelling, years of education, ETS exposure, BMI, history of respiratory disease, meat consumption, fruit consumption, vegetable consumption, frequency of cooking, and smoking status, excluded fruit consumption when stratified by fruit consumption.

^b^Adjusted for age, type of dwelling, years of education, ETS exposure, BMI, history of respiratory disease, meat consumption, fruit consumption, vegetable consumption and frequency of cooking, excluded fruit consumption when stratified by fruit consumption.

^c^The cutoff for low and high fruit consumption was determined by the median weekly servings of fruits that controls reported (7.36 servings/week). Bold values refer to statistically significant results with *P*<0.05.

The interaction effect between family history of any cancer and fruit consumption was statistically significant (*P* = 0.021). The cutoff for low and high fruit consumption was determined by the median weekly servings of fruits among controls (7.36 servings/week). When stratified by fruit consumption level, both low and high fruit consumption groups who had family history of lung cancer were associated with a higher risk of lung cancer among the overall population (aOR 1.98, 95% CI 0.97–4.06 and aOR 2.24, 95% CI 1.05–4.77, respectively) and never-smokers (aOR 3.09, 95% CI 1.37–7.01 and aOR 2.58, 95% CI 1.14–5.84, respectively) (Table 3). Besides, there was a significant association observed for those who had family history of any cancer among the low fruit consumption group in the overall population (aOR 1.88, 95% CI 1.24–2.86). Restricting to never-smokers only, this association was still significant (aOR 1.99, 95% CI 1.24–3.19).

## Discussion

This study examined the association between family history of lung cancer and lung cancer risk among Chinese women in Singapore using data from a case–control study. We found a significantly higher risk of lung cancer among Singaporean Chinese women who had an affected first-degree relative with lung cancer, especially among never-smokers, for both low and high levels of fruit consumption groups.

In our study, family history of lung cancer is a significant risk factor of lung cancer. This is consistent with previous studies that reported ORs of between 1.48 to 1.89 for the association between family history of lung cancer and lung cancer risk among women^15,19,20^. However, this cannot be immediately attributed to the heritability of genes that cause lung cancer susceptibility, as it may be possible to attribute familial aggregation of lung cancer to shared lifestyle habits as well. Studies have shown that smoking habits in children were highly correlated with smoking habits in parents^21,22^. Since smoking is the most well-established risk factor for lung cancer, family composition that includes heavy smokers may explain familial clustering of lung cancer cases. Therefore, we examined women who were never-smokers to minimize smoking as a confounding factor and found similar significant associations.

Our stratified analysis by smoking status showed that family history of lung cancer as a risk factor is more pronounced among never-smokers, with a significant OR of 2.78 (95% CI 1.57–4.90). This suggests that familial clustering of lung cancer is possibly not due to shared familial smoking habits among Singaporean women. It is also congruous with our finding of a significant interaction between smoking status and family history of lung cancer. Similarly, this interaction has been reported in some previous studies restricted to Caucasian women^23,24^. Furthermore, the observed OR in our study is close to the value reported in two prior meta-analyses that were restricted to studies conducted among never-smoking women in China. The most recent study conducted by Yu et al. (2016), included seven studies in its meta-analysis of familial aggregation of lung cancer, and elucidated an overall OR of 2.45 (95% CI 1.80–3.34)^16^. The other was conducted by Zhang et al. (2001) comprising three studies found a similar overall OR of 2.79 (95% CI 2.22–3.48)^25^. Our findings are comparable to previous studies, and adds to the existing body of evidence that family history of lung cancer is a significant risk factor among never-smoking women of Chinese ethnicity.

A family history of any cancer was also a significant risk factor in both the overall population and never-smokers in this study. The OR for family history of any cancer was found to be lower compared with that for family history of lung cancer, a trend consistent among both the overall population and never-smokers. A similar trend is seen in the aforementioned meta-analysis by Yu et al., where the OR for family history of any cancer among Chinese female never-smokers was 2.02 (95% CI 1.67–2.44), in comparison with the OR for family history of lung cancer which was 2.45 (95% CI 1.80–3.34)^16^. Unexpectedly, we did not observe any significant associations between family history of lung/any cancer and lung cancer among ever-smokers in our study. This is in contrast to previous studies that found lung cancer risk related to family history of lung cancer was higher among female smokers or ex-smokers compared with female never-smokers^26,27^. Our findings among ever-smokers should be interpreted with caution in light of the relatively small number of ever-smokers in our study population.

According to previous studies, some dietary factors were significantly associated with cancer risk among those with a family history of cancer in first-degree relatives^28,29^, but some studies showed no interactions between family history and dietary factors^30^. Dietary factors may potentially alter gene expression via epigenetic mechanisms^31^. For example, a Western pattern diet, characterized by high intakes of red and processed meat, fried foods, high-fat dairy products and high-sugar drinks, was found to affect histone polyacetylation and reduce short-chain fatty acids, leading to altered hepatic gene expression^32^. In our study, when restricted to never-smoking women, a significant association between family history of any cancer and a higher risk of lung cancer was observed among those who had low fruit consumption. Results from a previous study found that higher fruit consumption had a protective effect against lung cancer among never-smoking women^18^. A prior meta-analysis also showed that every 100 g/day increase in fruit consumption reduces lung cancer risk by 8%^33^. This protective effect could be driven by various beneficial compounds in fruits such as flavonoids combating oxidative stress that leads to carcinogenesis^34^. However, it is worthwhile to note that even among the high fruit consumption group in our study, the lung cancer risk was still elevated, suggesting that the protective effect of fruit consumption may be outweighed by having a family history of lung cancer. Besides, a few studies have implemented healthy lifestyle interventions to a high-risk stratum based on individuals with a family history of the disease, such as medical recommendations - to eat more healthily and stop smoking - although the effectiveness was limited^35,36^.

Consistent with the existing body of knowledge, we found a slightly elevated risk for NSCLC and adenocarcinoma. A pooled case–control study reported an OR of 1.58 for NSCLC (95% CI 1.44–1.73) and an OR of 1.59 for adenocarcinoma (95% CI 1.45–1.74)^15^, and a meta-analysis of nine studies reported an OR for adenocarcinoma of 1.60 (95% CI 1.32–1.93)^20^. These results complement the observation that adenocarcinoma is more prevalent among never-smokers than other histologic subtypes such as SCLC and squamous cell carcinoma^37^. As the relative risk associated with smoking for adenocarcinoma is lower than other histologic subtypes^38^, it has been hypothesized that adenocarcinoma is associated with other risk factors^37^. In this study, we found that the association between family history of lung cancer and adenocarcinoma was higher among never-smokers than in the overall population. Hence, it is possible that family history of lung cancer is one of the contributing risk factors to adenocarcinoma development in Singaporean never-smoking women. As there were too few non-adenocarcinoma NSCLC cases in the study, we were unable to conduct further stratified analysis by lung cancer subtypes. A previous meta-analysis by Lissowska et al. and pooled analysis by Cote et al. showed that squamous cell carcinoma and other subtypes of NSCLC were associated with family history^15,20^. However, further studies with a larger population with sufficient statistical power will be needed to ascertain if other NSCLC subtypes are associated with family history of lung cancer in Singapore.

This study has several strengths. To our best knowledge, this is the first study in a Southeast Asian population to investigate the association between family history and lung cancer risk. Even though there has been literature published on risk factors for lung cancer in Singaporean women^17,18,39^, there is still much to be studied about this population. Furthermore, this study population was unique in that it had a high proportion of never-smokers among lung cancer cases (67.1%). This allowed us to conduct analysis restricted to never-smokers, even when stratified by other risk factors, while retaining appreciable statistical power.

However, the study has several limitations. Due to the self-reported nature of the data, there is a possibility of recall bias. It is possible that lung cancer cases remembered their exposures better as compared to controls. However, a previous review found that self-reporting of family history of lung cancer has enough validity to be useful in epidemiological research^40^. Therefore, this should not affect the validity of this study. Secondly, the information collected on family history in the original questionnaire was limited. For example, we did not collect information on the number of family members diagnosed with lung cancer. Previous studies have shown that lung cancer risk increases with an increasing number of family members with lung cancer^41^. As we did not have this data, we are unable to see if this holds true in the Chinese Singaporean female population. Thirdly, there were only a small number of ever-smokers in this study as only 3–4% of Singaporean women are smokers^8^. Therefore, a stratified analysis among ever-smokers could not be conducted due to the lack of statistical power. Finally, for non-NSCLC histologic subtypes, there were too few subjects to calculate a meaningful OR. 

## Conclusions

In this paper, we found a significant, positive association between family history of lung cancer and lung cancer risk among Singaporean Chinese women. Lung cancer risk was further elevated among never-smoking women. Future studies are warranted to assess this association in other ethnicities and lung cancer subtypes.

## Supplementary Information

Supplementary Information.

## Data Availability

The data that support the findings of this study are available on request from the corresponding author. The data are not publicly available due to privacy or ethical restrictions.
