# Supplementary material for: Association between family history and lung cancer risk among Chinese women in Singapore
Source: Sci Rep. 2021 Nov 8;11:21862. doi: 10.1038/s41598-021-00929-9 (PMC8575905; doi:10.1038/s41598-021-00929-9)
Supplement: Supplementary file 1 — Supplementary Information. [file 41598_2021_929_MOESM1_ESM.pdf]

Supplementary Information

Supplementary Table 1. Histological subtypes of lung cancer

Histologic type	ICD-O-3 codes
Non-small cell lung cancer	• 8070/3 (Squamous cell carcinoma, NOS)<br>• 8012/3 (Large cell carcinoma, NOS)<br>• 8046/3 (Non-small cell carcinoma)<br>• 8140/3 (Adenocarcinoma, NOS)<br>• 8260/3 (Papillary adenocarcinoma, NOS)<br>• 8480/3 (Mucinous adenocarcinoma)<br>• 8250/3 (Lepidic adenocarcinoma)<br>• 8253/3 (Adenocarcinoma of lung, mucinous)
Adenocarcinoma	• 8140/3 (Adenocarcinoma, NOS)<br>• 8260/3 (Papillary adenocarcinoma, NOS)<br>• 8480/3 (Mucinous adenocarcinoma)<br>• 8250/3 (Lepidic adenocarcinoma)<br>• 8253/3 (Adenocarcinoma of lung, mucinous)
Squamous cell carcinoma	• 8070/3 (Squamous cell carcinoma, NOS)
Large cell carcinoma	• 8012/3 (Large cell carcinoma, NOS)
Unspecified NSCLC	• 8046/3 (Non-small cell carcinoma)
Small cell lung cancer	• 8041/3 (Small cell carcinoma, NOS)
Neuroendocrine carcinoma	• 8013/3 (Large cell neuroendocrine carcinoma)<br>• 8240/3 (Neuroendocrine tumour, NOS)<br>• 8246/3 (Neuroendocrine carcinoma, NOS)

Supplementary Table 2. Distribution of histologic type of lung cancer among cases and controls with and without family history of lung cancer

	**All women**	
**Family history (FH) of lung cancer in first-degree relatives**	**Cases (n (%))**	**Controls (n (%))**
**Non-adenocarcinoma non-small cell lung cancer**	(n=54)	(n=650)
FH absent	47 (87.0%)	608 (93.5%)
FH present	7 (13.0%)	42 (6.5%)
**Small cell lung cancer**	(n=17)	(n=650)
FH absent	14 (82.4%)	608 (93.5%)
FH present	3 (17.6%)	42 (6.5%)
**Neuroendocrine carcinoma**	(n=4)	(n=650)
FH absent	2 (50.0%)	608 (93.5%)
FH present	2 (50.0%)	42 (6.5%)

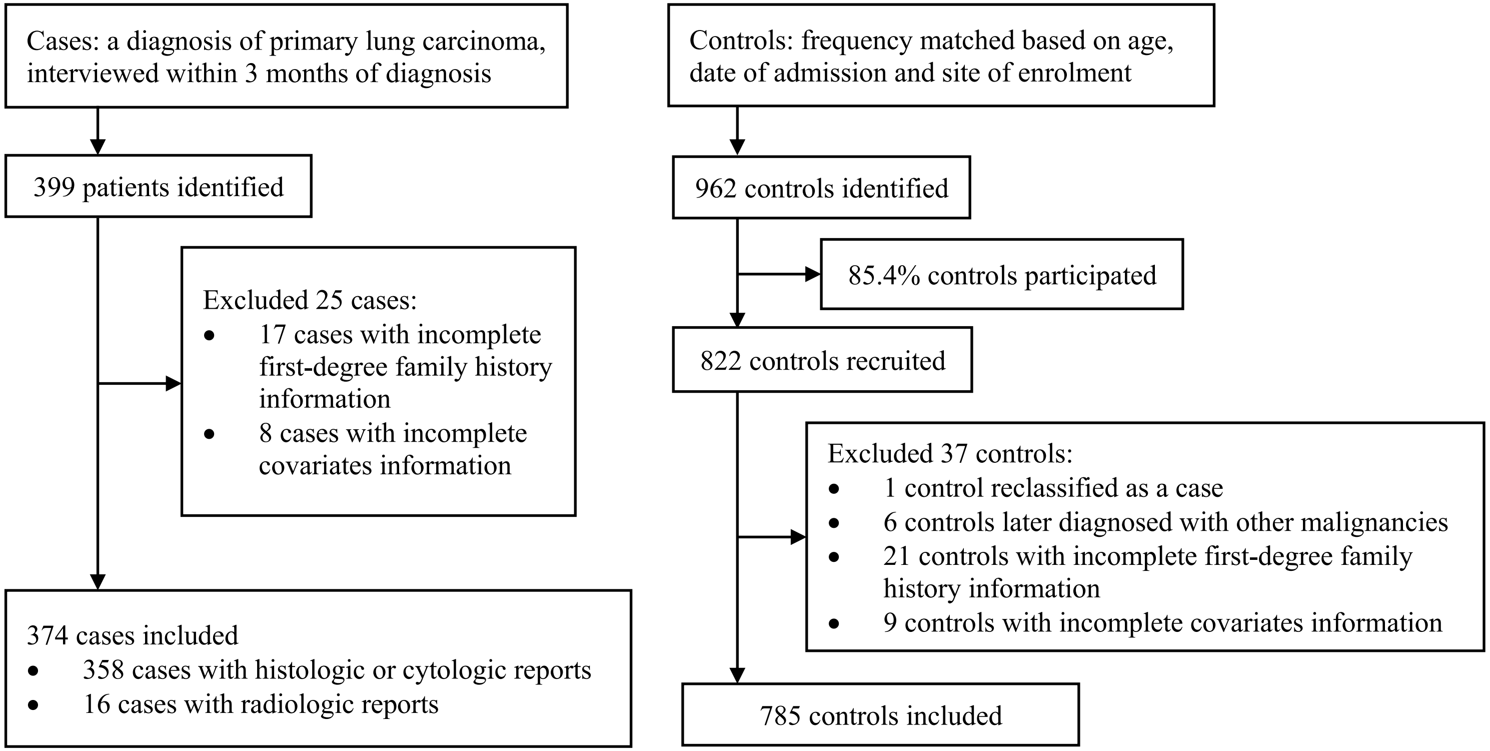

Supplementary Figure 1. Flowchart of study inclusion and exclusion criteria
